# Micropropagation and Quantification of Bioactive Compounds in *Mertensia maritima* (L.) Gray

**DOI:** 10.3390/ijms20092141

**Published:** 2019-04-30

**Authors:** Han Yong Park, Doo Hwan Kim, Ramesh Kumar Saini, Judy Gopal, Young-Soo Keum, Iyyakkannu Sivanesan

**Affiliations:** 1 Department of Bioresource Engineering, Sejong University, 98 Gunja-dong, Gwangjin-gu, Seoul 143-747, Korea; hypark@sejong.ac.kr; 2 Department of Bioresources and Food Science, Konkuk University, 1, Hwayang-dong, Gwangjin-gu, Seoul 05029, Korea; kimdh@konkuk.ac.kr (D.H.K.); judy.je.gopal@gmail.com (J.G.); 3 Department of Crop Science, Konkuk University, 1, Hwayang-dong, Gwangjin-gu, Seoul 05029, Korea; saini_1997@yahoo.com (R.K.S.); rational@konkuk.ac.kr (Y.-S.K.)

**Keywords:** carotenoids, cytokinins, γ-linolenic acid, micropropagation, stearidonic acid, tocopherol

## Abstract

The goal of this study was to establish an efficient protocol for the large-scale propagation of *Mertensia maritima* (L.) Gray, and evaluate the carotenoid, fatty acid, and tocopherol contents in the leaves of in vitro regenerated shoots. Surface-disinfected node and shoot tip explants were placed on semisolid Murashige and Skoog (MS) medium with 0–16 µM N^6^-benzyladenine (BA), kinetin, (KN), and thidiazuron (TDZ) alone, or in combination with, 1 or 2 µM α-naphthaleneacetic acid (NAA). Of the three different cytokinins employed, TDZ elicited the best results for axillary shoot proliferation. A maximum frequency of shoot initiation above 84%, with a mean of 8.9 and 4.8 shoots per node and shoot tip, respectively, was achieved on the culture medium supplemented with 4 µM TDZ. A combination of TDZ + NAA significantly increased the percentage of multiple shoot formation and number of shoots per explant. The best shoot induction response occurred on MS medium with 4 µM TDZ and 1 µM NAA. On this medium, the node (93.8%) and shoot tip (95.9%) explants produced an average of 17.7 and 8.6 shoots, respectively. The highest root induction frequency (97.4%) and number of roots per shoot (25.4), as well as the greatest root length (4.2 cm), were obtained on half-strength MS medium supplemented with 4 µM indole-3-butyric acid (IBA). The presence of six carotenoids and α-tocopherol in the leaf tissues of *M. maritima* was confirmed by HPLC. Gas chromatography-mass spectrometry analysis confirmed the presence of 10 fatty acids, including γ-linolenic acid and stearidonic acid in the leaf tissues of *M. maritima*. All-*E*-lutein (18.49 μg g^−1^ fresh weight, FW), α-tocopherol (3.82 μg g^−1^ FW) and α-linolenic acid (30.37%) were found to be the significant compounds in *M. maritima*. For the first time, a successful protocol has been established for the mass propagation of *M. maritima* with promising prospects for harnessing its bioactive reserves.

## 1. Introduction

Micropropagation is widely used technique for germplasm conservation and extensive propagation of various plants. This method has also been employed in several biotechnological applications including the production of bioactive compounds [1]. In recent years, several bioactive compounds like alkaloids, anthocyanins, carotenoids, fatty acids, flavonoids, phenolic acids, and tocopherols have been obtained from in vitro-developed shoots [2,3,4,5,6,7]. The synthesis and accumulation of specific bioactive compounds in shoot cultures depend largely on the composition of culture media and the environmental conditions of the culture [4,6]. Carotenoids, flavonoids, phenolic acids and tocopherols are potent antioxidants, and are reported to possess numerous additional biological activities [8,9].

*Mertensia maritima* (L.) Gray (Boraginaceae), commonly known as the oyster plant, is a perennial herb native to northern Europe, including the British Isles [10]. It inhabits shingle beaches, and rarely sandy beaches, and is difficult to cultivate in the garden because of stringent temperature requirements [11]. *Mertensia maritima* has been grown in Northern Scotland and Southwestern France for its fragrant leaves [12]. The oyster plant is naturally propagated through seeds, but germination is poor due to seed dormancy. Indeed, Skarpaas and Stabbetorp [13] reported that a cold period was essential to break seed dormancy and a cold treatment of oyster plant seeds at 2 °C was shown to enhance germination. The food reserve in *M. maritima* is fat [11] and the volatile composition of the oyster plant has been well-documented [12], with approximately 109 volatile compounds being identified from *M. maritima* leaf extracts. In addition, three main compounds, allantoin, rabdosiin, and rosmarinic acid, were isolated from callus extracts of *M. maritima* [14]. However, there are no reports on the content and composition of carotenoids, fatty acids and tocopherols from tissues and organs of *M. maritima*.

There are several studies detailing the in vitro propagation of plant species in Boraginaceae including *Arnebia hispidissima* [15,16], *Cordia verbenacea* [17], *Hackelia venusta* [18], *Heliotropium indicum* [19], *Sericostoma pauciflorum* [20], and *Trichodesma indicum* [21,22]. However, to the best of our knowledge, no report has been published on *in vitro* propagation of *Mertensia* species and for *M. maritima*, only the callus induction protocol has been developed using leaf and apical shoot explants [14]. The goal of this study was to develop an efficient and rapid system for the micropropagation of *M. maritima*, which would be expected to overcome current propagational barriers. Furthermore, we also investigated the bioactive reservoirs such as carotenoid, tocopherol and fatty acid contents of in vitro-derived shoots. This is the first report in this respect.

## 2. Results 

### 2.1. Micropropagation

**Figure 1:**
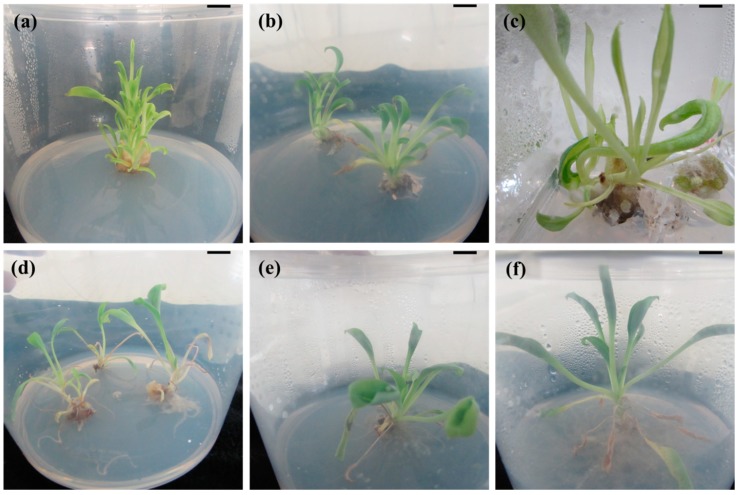
In vitro propagation of *Mertensia maritima*. Multiple shoot induction from node (**a**) and shoot tip (**b**) explants grown on Murashige and Skoog (MS) medium with 4 µM thidiazuron (TDZ) and 1 µM α-naphthaleneacetic acid (NAA); (**c**) hyperhydric shoots of *M. maritima* on MS medium with 12 µM TDZ and 2 µM NAA; (**d**) rooting on half-strength MS medium supplemented with 12 µM indole-3-butyric acid (IBA); rooting on half-strength MS medium supplemented with 4 µM IBA after (**e**) four weeks and (**f**) 8 weeks of culture. Scale bar: 2 cm.

Node and shoot tip explants of *M. maritima* failed to develop shoots after four weeks of culture on MS (control) medium. Conversely, shoots were produced within a week from both node and shoot tip explants when they were cultured on semisolid MS medium supplemented with N^6^-benzyladenine (BA), kinetin (KN), or thidiazuron (TDZ). However, the shooting response of the explants was dependent on the type and levels of the cytokinins (Table 1). Of the two explants studied, the node was more responsive to multiple shoot production than the shoot tip (*p* < 0.05). Of the various levels of BA (1–16 µM) studied, the best response was observed on semisolid MS medium with 8 µM BA, with the node and shoot tip explants producing 4.4 and 2.6 shoots, respectively. Increasing levels of BA ≥8 µM reduced the average number of shoots in both explants. Among the six different concentrations of KN used, the best response was obtained on MS medium with 8 µM KN, with node (58.6%) and shoot tip (65.4%) explants producing a mean of 5.2 and 3.4 shoots, respectively (Table 1). Increasing concentrations of KN ≥8 µM reduced both the rate of shoot induction and average number of shoots in both types of explants. Supplementing the growth medium with TDZ at 1–16 µM significantly (*p* < 0.05) promoted shoot initiation; however, the shoot induction percentage and the average number of induced shoots per node or shoot tip were highly affected by TDZ concentration. A maximum frequency of shoot initiation, above 84% and with an average of 8.9 and 4.8 shoots per node and shoot tip, respectively, was achieved on semisolid MS medium supplemented with 4 µM TDZ (Table 1), but the shooting response was reduced with higher levels of TDZ 8–16 µM. A combination of TDZ + NAA significantly (*p* < 0.05) increased the shooting response of both explants, as well as the average number of shoots developed per explant (Table 2). The best shoot induction response was found on semisolid MS medium with 4 µM TDZ and 1 µM NAA (Figure 1a,b), with node (93.8%) and shoot tip (95.9%) explants producing an average of 17.7 and 8.6 shoots, respectively. The addition of 2 µM NAA to TDZ-containing medium significantly (*p* < 0.05) decreased the number of shoots produced in both node and shoot tip explants of *M. maritima* (Table 2). Hyperhydric shoots developed on semisolid MS medium supplemented with 8–16 µM TDZ and 1–2 µM NAA (Figure 1c).

The regenerated shoots developed roots within two weeks when grown on half-strength MS medium with or without auxin. Only 26% of shoots rooted on auxin-free medium after 56 days of culture. Among the different IAA, IBA and NAA concentrations used, 8, 4, and 4 µM, respectively, were found to be the ideal levels for rooting of *M. maritima* shoots. Increasing concentrations of auxin above the optimal level decreased the percentage of root formation, as well as the mean number and length of the roots. High auxin levels 8–12 µM induced leaf senescence and callus formation at the shoot base (Figure 1d). Of the three different auxins employed, IBA elicited the best rooting results. The highest root induction frequency (97.4%) and number of roots per shoot (25.4), as well as the greatest root length (4.2 cm), were achieved on the half-strength semisolid MS medium supplemented with 4 µM IBA (Table 3, Figure 1e,f). The well-developed rooted plantlets (56 days old) were acclimatized in a greenhouse.

### 2.2. Content of Bioactive Compounds

**Figure 2:**
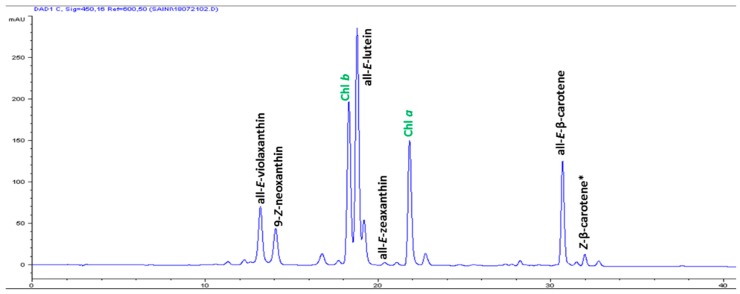
High-performance liquid chromatograms (HPLCs) (450 nm) of carotenoids in the leaf tissues of *Mertensia maritima*. Chl: chlorophyll.

**Figure 3:**
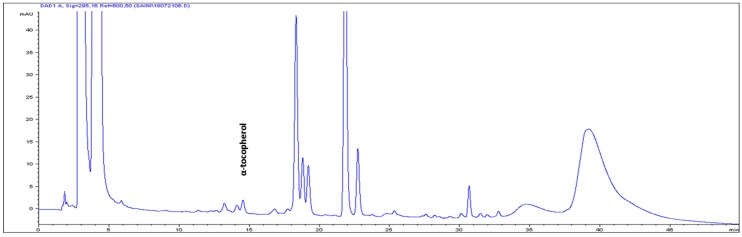
High-performance liquid chromatograms (HPLCs) (295 nm) of α-tocopherol in the leaf tissues of *Mertensia maritima*.

**Figure 4:**
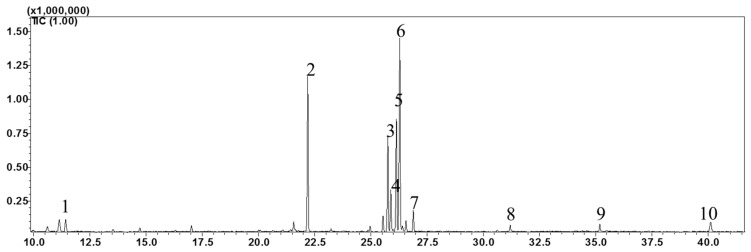
Gas chromatogram (GC) of fatty acid methyl esters (FAMEs) in the leaf tissues of *Mertensia maritima*. The following FAME were identified: (**1**) lauric acid (Retention Time (RT): 11.42 min); (**2**) palmitic acid (RT: 22.195 min); (**3**) γ-linolenic acid (RT: 25.755 min); (**4**) stearidonic acid (RT: 25.89 min); (**5**) linoleic acid (RT: 26.135 min); (**6**) α-linolenic acid (RT: 26.29 min); (**7**) stearic acid (RT: 26.89 min); (**8**) arachidic acid (RT: 31.2 min); (**9**) behenic acid (RT: 35.185 min); and (**10**) lignoceric acid (RT: 40.12).

**Figure 5:**
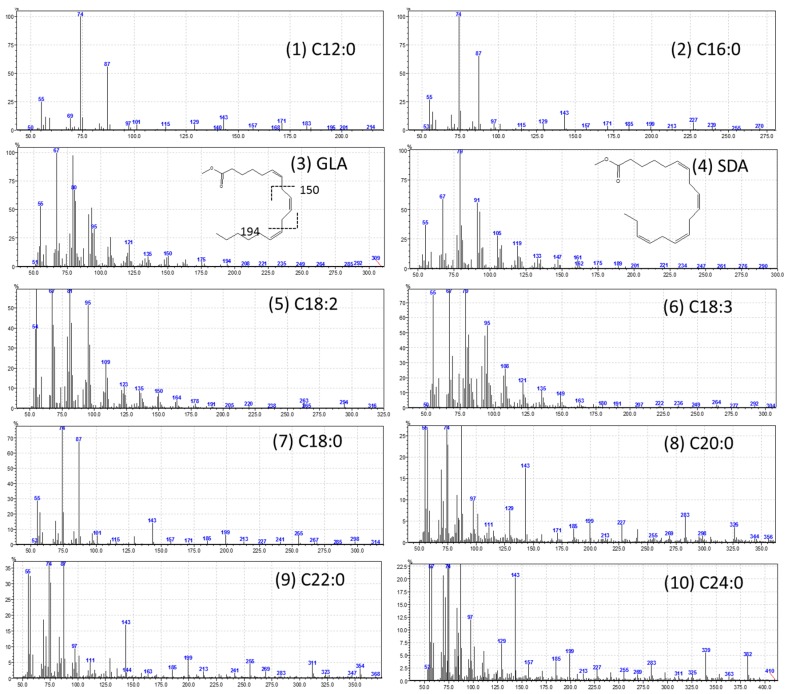
Mass spectra of fatty acid methyl esters (FAMEs). (**1**) C12:0 (lauric acid); (**2**) C16:0 (palmitic acid); (**3**) C18:3n6 (γ-linolenic acid); (**4**) C18:4n3 (stearidonic acid); (**5**) C18:2n6c (linoleic acid); (**6**) C18:3n3 (α-linolenic acid); (**7**) C18:0 (stearic acid); (**8**) C20:0 (arachidic acid); (**9**) C22:0 (behenic acid); and (**10**) C24:0 (lignoceric acid).

The high-performance liquid chromatography (HPLC) analysis confirmed the presence of six carotenoids and α-tocopherol in the leaf tissues of *M. maritima* (Figure 2 and Figure 3). All-*E*-lutein was the most abundant of the six carotenoids (18.49 μg g^−1^ fresh weight, FW), followed by all-*E*-β-carotene (6.42 μg g^−1^ FW), all-*E*-violaxanthin (5.59 μg g^−1^ FW), 9-*Z*-neoxanthin (3.44 μg g^−1^ FW), (*Z*)-β-carotene (0.74 μg g^−1^ FW) and all-*E*-zeaxanthin (0.29 μg g^−1^ FW) in the leaves obtained from in vitro-raised *M. maritima* shoots (Table 4). The total carotenoid content in the leaves of in vitro-raised oyster plants was 34.97μg g^−1^ FW. The α-tocopherol content in the leaf tissues of *M. maritima* was 3.82 μg g^−1^ FW (Table 4). The gas chromatography-mass spectrometry (GC-MS) analysis confirmed the presence of 10 fatty acids in the leaf tissues of *M. maritima* (Table 5, Figure 4 and Figure 5). Of the 10 fatty acids identified, α-linolenic was the most abundant (30.37%) in *M. maritima* leaf tissues followed by palmitic and linoleic primary fatty acids with 22.66% and 16.90%, respectively. Important fatty acids like γ-linolenic acid (GLA, 14.05%) and stearidonic acid (SDA, 6.04%) were also found in the leaves. Γ-linolenic acid and SDA were identified by comparing their fragmentation pattern, retention time with fatty acid methyl esters (FAMEs) standards and by comparison with the NIST library (Figure 4 and Figure 5). Stearic, lignoceric, behenic and arachidic were the minor fatty acids in the leaf tissues of *M. maritima* with values of 3.14, 2.44, 1.12, and 0.84%, respectively. Polyunsaturated fatty acids content (PUFAs, 67.36%) was higher than that of saturated fatty acids (SFAs 30.37%) in the leaf tissues of *M. maritima* (Table 5). The PUFA: SFA ratio was 2.06%. The total lipid content in the oyster plant leaf was 7.8%. The present study showed that oyster leaves contain a high amount of α-linolenic acid, GLA and SDA and therefore, can be considered a potential source of these bioactive compounds.

## 3. Discussion

Explants with vegetative meristems such as nodes and shoot tips are commonly used to obtain multiple shoots [23]. Shoot formation was not observed when node and shoot tip explants of the oyster plant were cultured on hormone-free growth medium, a similar result has been observed for other members of the Boraginaceae [18,22]. For example, shoot tip explants of *H. venusta* [18] and *T. indicum* [22] failed to grow on semisolid MS medium devoid of plant hormones. In this study, shoots developed from oyster plant explants cultured on semisolid medium containing cytokinins (Table 1), which are often included in medium to promote shoot initiation and multiplication. The stimulatory effect of cytokinins on axillary shoot induction has also been reported for other members of the Boraginaceae such as *C. verbenacea* [17], *H. venusta* [18], *H. indicum* [19], and *T. indicum* [22]. In micropropagation, BA and KN (adenine derivatives), and TDZ (a phenylurea derivative) are often used for shoot multiplication. In the present study, TDZ was the most benificial for shoot proliferation compared to KN and BA. The positive impact of TDZ on multiple shoot production has also been reported in several plants like *Aronia melanocarpa* [23], *Bacopa monnieri* [24], and *Jeffersonia dubia* [25]. In addition, several reports have confirmed that an auxin and cytokinin combination improves multiple shoot induction in members of the Boraginaceae [15,17,19,22]. Lameira and Pinto [17] reported that 5 µM KN in combination with 0.01 µM NAA improved multiple shoot induction from apical segments (2.7) of *C. verbenacea*. Moreover, a combination of 4.7 µM KN, 2.2 µM BA and 0.28 µM IAA had a positive effect on shoot production in the apical (32.6) and axillary (20.2) buds of *Heliotropium* indicum explants [19]. In *A. hispidissima*, the highest rate of shoot induction was obtained from shoot tips (93%) and nodes (60%) cultured on MS medium supplemented with 2.3 µM KN, 2.2 µM BA and 0.57 µM IAA [15] and Mahesh and Jeyachandran [22] reported that 4.4 µM BAP in combination with 2.69 µM NAA improved multiple shoot induction (9.94) from *T. indicum* shoot tip explants. In the present study, a significantly greater number of shoots were obtained in both explant types grown on semisolid MS medium with 4 µM TDZ and 1 µM NAA (Table 2), and similar results have been observed in several other plants [23,25,26].

In this study, hyperhydric (deformed) shoots were induced on growth medium supplemented with high levels of TDZ alone or with TDZ plus NAA (Figure 1c). Hyperhydricity is an anatomical, morphological and physiological disorder that frequently affects the commercial in vitro micropropagation of many plants. Shoots developed on semisolid MS medium containing 8–16 µM TDZ plus 1 or 2 µM NAA were fragile, translucent, turgid, and wrinkled (Figure 1c). Therefore, low levels of TDZ (2 or 4 µM) plus 1 µM NAA are recommended for shoot production in *M. maritima*. High TDZ concentrations (above the optimal level) and/or constant exposure to TDZ have been reported to result in hyperhydric shoots that exhibit reduced growth, and may even die, when transferred to the same growth medium for a subsequent passage [27,28,29]. Auxin is regularly added to culture medium to stimulate root induction in regenerated shoots of members of the Boraginaceae [15,16,18,19,20,22,30]. The half-strength semisolid MS medium with 8 µM IAA, 4 µM IBA or 4 µM NAA presented 68.8, 97.4 or 66.2% rooting success (Table 3). The superior effect of IBA on rooting has also been reported for *A. hispidissima* [15,16], *H. venusta* [18], and *T. indicum* [21,22].

Carotenoids are naturally occurring organic pigments synthesized by algae, bacteria, fungi, and plants [31] and are vital nutrients for both animals and humans. To our knowledge, the carotenoid composition and total carotenoid contents in the organs of *M. maritima* have not been previously reported. All-*E*-lutein was the major carotenoid in leaf tissues (from *in vitro*-raised shoots on MS + 4 µM TDZ + 1 µM NAA) of *M. maritima* and a similar result has been reported for the leaves of *Ajuga multiflora* [3] and *Aronia melanocarpa* [23] and the shoots of *Sedum dasyphyllum* [32]. All-*E*-lutein has also been identified as the predominant carotenoid in the leaf extracts of another member of the Boraginaceae, *Cordia myxa* [33]. The tentative identification of (*Z*)-β-carotene (λmax—446 and 474 nm) was based on the HPLC-PDA spectral and chromatographic behavior reported in the literature [34] and quantified according to the external standard curve of all-*E*-β-carotene, due to their relatively similar spectrophotometric absorption properties [35]. The total carotenoid content in the leaves of in vitro-raised oyster plants was 34.97 μg g^−1^ FW higher compared to that of flower extracts (0.054 μg g^−1^ dry weight (DW), or ≈0.0054 μg g^−1^ FW considering 90% moisture) of *Borago officinalis* [36]. In the present study, HPLC analysis only confirmed the presence of α-tocopherol (Figure 3), whereas the other forms of tocopherol were not detected in the oyster plant leaves. The beneficial effects of all-*E*-lutein, all-*E*-β-carotene and α-tocopherol on human health are well documented [37,38].

Poly-unsaturated fatty acids like linoleic, α-linolenic, γ-linolenic, and stearidonic acids are important for human health and nutrition [39] and are also beneficial for plant abiotic stress tolerance, like cold acclimation. In this study, high amounts of PUFAs were recorded in the oyster plant leaf (from in vitro-raised shoots on MS + 4 µM TDZ + 1 µM NAA). Although *M. maritima* is a hardy plant, the increased accumulation of PUFAs in the leaves of regenerated shoots may have been due to the stressful in vitro conditions. GLA and SDA are unusual PUFAs, which have potential cosmetic, medicinal, and nutritional applications [39]. Both GLA and SDA are Δ6-desaturation products derived from linoleic and α-linolenic acids, respectively. The GC-MS analysis identified the presence of GLA and SDA in oyster plant leaf. The important fatty acids GLA and SDA have also been found in other members of the Boraginaceae [40,41,42]. The high level of GLA is found usually in seed oils of several plant species. It is also found in various organs such as leaves, flowers, stems and roots of some plant species [43,44,45,46,47]. However, the synthesis and accumulation of GLA in organs largely depend on plant species, genotype, growth stage, and environmental factors. In the present study, a considerable level of GLA was found in *M. maritima*. The GLA content in the leaf tissues (14.05%) of in vitro-developed shoots was also greater than that found in the leaves of *Borago officinalis* (2.5–6.5%), *Delphinium gracile* (0.033%), *Epilobium hirsutum* (1.4%), *Epilobium lanceolatum* (0.81%), *Myosotis nemorosa* (1.45%), *Primula cortusoides* (4.0%), *Primula denticulata* (5.9%), *Primula farinosa* (5.4%), *Primula glaucescens* (6.0%), *Primula luteola* (1.9%), *Primula marophylla* (5.7%), *Primula malacoides* (10.9%), *Primula scotica* (6.7%), *Primula vulgaris* (0.6%), *Primula vialii* (1.5%), and *Scrophularia sciophila* (0.3%); stems of *Borago officinalis* (10.1–11.5%), *Cynoglossum creticum* (4.07%), *Scrophularia sciophila* (0.7%); roots of *Borago officinalis* (13.7%), *Cynoglossum creticum* (7.16%), *Echium creticum* (2.9%), *Primula cortusoides* (1.9%), *Primula denticulata* (0.2%), *Primula farinosa* (0.1%), *Primula glaucescens* (0.3%), *Primula luteola* (0.6%), *Primula marophylla* (0.4%), *Primula malacoides* (1.4%), *Primula scotica* (0.2%), *Primula vulgaris* (0.8%), *Primula vialii* (0.1%), *S. sciophila* (0.27%); and flowers of *Anchusa azurea* (3.56%), and *Scrophularia sciophila* (0.28%) [43,44,45,46,47].

The stearidonic acid is mainly obtained from the seed oils of blackcurrant, borage, echium, and hemp [48]. SDA has also been detected in leaves, stems and roots of some plant species [40,44,45,48,49]. However, SDA is found at low levels in the vegetarian diet. Therefore, finding of new leafy vegetables containing higher amounts of SDA have great interest. In this study, a considerable level of SDA was found in *M. maritima*. Thus, oyster plant can be considered as potential source of leafy vegetable for cultivation. The SDA content in leaf tissues (6.04%) of the oyster plant was higher compared to that found in the leaves of *Cynoglossum creticum* (2.76%), *Scrophularia sciophila* (1.36%); stems of *Cynoglossum creticum* (4.12%), *Scrophularia sciophila* (0.59%); roots of *Primula denticulata* (3.3%), *Primula malacoides* (1.5%), *Primula scotica* (4.0%), *Primula vialii* (4.0%), and *Scrophularia sciophila* (0.29%); flowers of *Cynoglossum creticum* (5.46%), and seed oil of *Borago longifolia* (0.3%), *Borago morisiana* (1.9%), *Borago pygmaea* (1.2%), *Borago trabutii* (0.3%), *Cynoglossum creticum* (2.1%), *Nonea vesicaria* (0.3%), *Nonea pulla* (3%), and *Pulmonaria officinalis* (2.5%) [40,44,45,48,49]. Unusual fatty acids, such as GLA and SDA, are not reported in most leafy vegetables. The presence of high concentrations of GLA and SDA in oyster plant leaves suggests this plant may be a potential source of these unusual fatty acids and the high levels of PUFAs in oyster plant leaves supports its nutritional value.

## 4. Methods

### 4.1. Micropropagation

Shoots of *M. maritima* were collected from three-month-old plants grown in a greenhouse and were surface-disinfected in 70% (*v*/*v*) ethanol for 90 s, and immersed in 2.0% (*v*/*v*) sodium hypochlorite (NaClO) containing two drops of Tween 20 for 10 min; the shoots were then washed with sterile distilled water 3–5 times for 2 min, and old leaves were removed. The shoots were again surface- disinfected with 70% (*v*/*v*) ethanol for 90 s, and 2.0% (*v*/*v*) NaClO for 10 min. The shoots were washed 3–4 times with sterile distilled water after ethanol and NaClO treatment. Node (0.5 cm) and shoot tip (1.0–1.5 cm) explants were prepared from the disinfected shoots and placed on semisolid Murashige and Skoog (MS) medium. The medium comprised MS nutrients, vitamins [50], 3% (*w*/*v*) sucrose and 0.8 % (*w*/*v*) plant agar. Plant hormones (N^6^-benzyladenine (BA), 6-furfurilaminopurine (kinetin, KN), indole-3-acetic acid (IAA), indole-3-butyric acid (IBA), and α-naphthaleneacetic acid (NAA)) were added to the MS medium before pH adjustment (5.8) using 0.1 N NaOH or HCl and sterilization (121 °C and 1.06 kg/cm^2^ for 20 min). Thidiazuron (1-phenyl-3-(1,2,3,-thiadiazol-5-yl)urea, (TDZ)) was filter-sterilized and added to the autoclaved medium.

The explants were placed on semisolid MS medium supplemented with various levels of BA, KN or TDZ 0–16 µM and 1–16 µM TDZ plus 1 or 2 µM NAA for shoot induction. For each treatment, 25 explants were used, with three replications. The explants were assessed for shoot induction after four weeks. Regenerated shoots (2–3 cm) were separated from the shoot bunches obtained from the ideal shoot induction medium (MS + 4 µM TDZ + 1 µM NAA) and cultured on half-strength MS medium supplemented with 0–12 µM IAA, IBA or NAA for root induction. For each treatment, 25 explants were used, with three replications. The rooting response of shoots was recorded after 56 days. The cultures were maintained at 23 ± 2 °C under a 16-h photoperiod with a light intensity of 60 µM m^−2^ s^−1^.

### 4.2. Extraction and Quantification of Carotenoids and Tocopherols

The carotenoids and tocopherols in the leaf tissues of *M. maritima* were extracted and quantified according to previously established protocols, with slight modifications [3]. Briefly, a 2 g leaf sample (from in vitro-raised shoots on MS + 4 µM TDZ + 1 µM NAA) was transferred into an amber glass vial with a pinch of magnesium carbonate and 10 mL of an acetone: hexane: ethanol (1:2:1) solution containing 0.1% (*w*/*v*) butylated hydroxytoluene (BHT). The samples were mechanically homogenized, bath sonicated (JAC-2010; 300 W, 60 Hz; Sonics & Materials Inc., Newtown, CT, USA) for 15 min; centrifuged at 5000× *g* (5 min at 4 °C) and the supernatant was recovered. The pelleted samples were continually extracted until the pellets became colorless. The supernatants were combined, vacuum-dried, redissolved in 1.0 mL of acetone containing 0.1% BHT, filtered through a nylon syringe filter (pore size 0.45 μm; Whatman, Amersham Place, Little Chalfont, Buckinghamshire, UK), and finally transferred to an amber-colored HPLC (High-performance liquid chromatography) vial. HPLC analysis was performed using an Agilent 1100 chromatograph (Agilent, Mississauga, ON, Canada) with an autosampler, a degasser, a diode array detector (DAD) set at 200–800 nm, and a dual pump. Carotenoids and tocopherols were separated using a YMC carotenoid C30 (250 × 4.6 mm, 5 μm) column (YMC, Wilmington, NC, USA) at 20 °C. The eluents were (A): methanol: methyl tertiary butyl ether: water (81:15:4) and (B): methyl tertiary butyl ether: methanol (91:9). The gradient solvent elution was 0–50 % B for 45 min, followed by 0% B and a 5 min post run at a flow rate of 1 mL min^-1^. A 20 µL sample was injected. DAD detection was measured at 295 and 450 nm for tocopherols and carotenoids, respectively.

### 4.3. Lipid Extraction and Fatty Acid Methyl Ester (FAME) Preparation

Lipids were extracted from dehydrated samples by our previously standardized method using chloroform-methanol extraction [51], based on the procedure reported by Bligh and Dyer [52]. Briefly, 1 g of finely powdered dehydrated oyster plant leaves (from in vitro-raised shoots on MS + 4 µM TDZ + 1 µM NAA) was homogenized with 20 mL of chloroform and methanol (2:1), followed by sonication (300 W, 60 Hz) for 15 min. Samples were centrifuged at 5000× *g* (5 min at 4 °C), the supernatant was recovered, and the pelleted samples were extracted again with 10 mL of chloroform and methanol (2:1). The supernatants from all the extractions were combined (total volume 50–70 mL) in a 250 mL separating funnel, partitioned with 20 mL of 0.85% (*w*/*v*) sodium chloride (NaCl). The lower organic (chloroform) phase was collected into a pre-weighed flask and dried in a rotary vacuum evaporator; the total lipid content was measured gravimetrically. Subsequently, FAMEs were prepared from extracted lipids by the conventional anhydrous methanolic hydrochloric acid (HCl) method [51]. Briefly, 3 mL of anhydrous methanolic HCl (5%, *v*/*v*) was added to the lipid sample in a 10 mL glass tube fitted with a Teflon-lined screw cap and incubated for 2 h at 60 °C in a water bath. After cooling, the FAME was sequentially washed with 5% NaCl and 2% sodium bicarbonate (NaHCO_3_) and recovered in 10 mL hexane. A pinch of anhydrous sodium sulfate (Na_2_SO_4_) was added to the recovered sample (hexane) to absorb the traces of water. Subsequently, 1 mL of the sample was filtered through a 0.45 µm PTFE syringe filter and transferred to a gas chromatography (GC) vial for analysis.

Fatty acid methyl esters (FAMEs) were analyzed using a GC-2010 Plus Gas Chromatograph (Shimadzu, Japan) equipped with an AOC-20 i Auto-injector and a GCMS-QP2010 SE Gas chromatography-mass spectrophotometer (MS) using an HP-5 column (Agilent; 30 m, 0.250 μm thick, and 0.25 mm ID). The injector port, ion source and interface temperatures were set at 250, 260 and 270 °C, respectively and helium was used as the carrier gas. First, the column temperature was maintained at 120 °C for 5 min, followed by an increase to 240 °C for 30 min, and then held at 240 °C for 25 min. The FAMEs were identified by comparing their fragmentation pattern and retention time with authentic standards (Supelco® 37 Component FAMES Mix, no. CRM47885) and with the NIST library.

The experiments were set up in a completely randomized design and with three replications. The experimental results were subjected to analysis of variance (ANOVA) using the SAS program (Release 9.4, SAS Institute, NC, USA). Data are expressed as the mean ± standard deviation (SD). The differences between the mean values were assessed by Duncan’s multiple range test (DMRT) at *p* < 0.05. 

## 5. Conclusions

In conclusion, for the first time, we have established an efficient protocol for the micropropagation of *M. maritima* using node and shoot tip explants. The inclusion of NAA in the TDZ-containing medium enhanced multiple shoot induction. Gas chromatography-mass spectrometry analysis revealed the presence of GLA and SDA in the leaves of *M. maritima*. The in vitro shoot culture may be useful for the production of GLA, as well as an excellent system for biochemical and molecular studies of GLA. The high frequency of multiple shoot induction and rooting suggests that this procedure can be used for mass propagation, thereby improving the value of this plant. 

## Figures and Tables

**Table 1:** Impact of cytokinins on multiple shoot induction in the node and shoot tip explants of *Mertensia maritima*.

Cytokinins (µM)	Shoot Induction (%)		Number of Shoots/Explant	
	Node	Shoot Tip	Node	Shoot Tip
0	0.0k	0.0m	0.0l	0.0h
BA 1	14.2j	24.7l	1.8k	1.2fg
2	24.5i	35.0k	2.8ij	1.7efg
4	44.4f	47.3h	3.3ghi	2.0def
8	49.1e	57.1fg	4.4cdef	2.6cd
12	57.1cd	59.4ef	3.0hij	2.3cde
16	37.6g	43.8i	2.3jk	1.4fg
KN 1	24.9i	34.8k	2.4ijk	1.3fg
2	32.7h	36.6jk	3.7fgh	1.9defg
4	38.4g	45.0hi	4.3def	2.6cd
8	58.6c	65.4d	5.2c	3.4b
12	54.7d	60.7e	4.0efg	3.0bc
16	47.7e	55.9g	3.2ghij	1.1g
TDZ 1	36.9g	38.5j	4.7cde	2.0def
2	65.7b	69.1c	6.1b	3.1bc
4	84.7a	87.0a	8.9a	4.8a
8	68.2b	72.7b	5.1cd	2.4cde
12	55.6cd	64.0d	2.6ijk	2.3cde
16	23.9i	25.9l	1.8k	1.7efg

Means followed by same letters (a–m) within a column are not significantly different according to Duncan’s multiple range test (*p* < 0.05). Coefficient of variation: Shoot induction (node—6.66, shoot tip—6.19), number of shoots per explant (node—21.62, shoot tip—35.37). Mean of nine replications.

**Table 2:** Effects of the combination of TDZ and NAA on multiple shoot induction from the node and shoot tip explants of *Mertensia maritima*.

TDZ (µM)	NAA (µM)	Shoot Induction (%)		Number of Shoots/Explant	
		Node	Shoot Tip	Node	Shoot Tip
1	1	49.2g	46.8f	6.8de	4.4d
2	1	80.3c	78.2d	10.8b	7.6b
4	1	93.8a	95.9a	17.7a	8.6a
8	1	77.8cd	85.0c	8.1c	4.6cd
12	1	66.3e	74.0e	4.8gh	2.9ef
16	1	29.8i	32.8g	3.8h	2.3f
1	2	42.6h	43.1f	5.1fg	2.0fg
2	2	70.2e	73.9e	7.8cd	3.8de
4	2	89.4b	91.1b	11.7b	5.4c
8	2	74.4d	78.1d	6.2ef	2.7f
12	2	61.3f	70.9e	2.1i	2.2f
16	2	28.4i	30.8g	1.3i	1.1g

Means followed by same letters (a–i) within a column are not significantly different according to Duncan’s multiple range test (*p* < 0.05). Coefficient of variation: Shoot induction (node—6.97, shoot tip—6.27), number of shoots per explant (node—18.21, shoot tip—25.23). Mean of nine replications.

**Table 3:** Impacts of concentrations of auxins on *in vitro* rooting of *Mertensia maritima*.

Auxins (µM)	Root Induction (%)	No. of Roots/Shoot	Mean Root Length (cm)
0	26.0j	3.4j	0.9i
IAA 2	47.9h	5.2hi	1.4h
4	60.8f	9.7fg	1.9g
8	68.8e	11.0e	2.4e
12	55.2g	6.7h	2.1fg
IBA 2	72.2d	15.0c	3.1c
4	97.4a	25.4a	4.2a
8	90.9b	20.7b	3.8b
12	84.3c	11.4e	2.8cd
NAA 2	58.3f	5.9hi	1.8g
4	66.2e	13.1d	2.8d
8	46.4h	8.4g	2.3ef
12	35.7i	4.9ij	1.4h

Means followed by the same letters (a–j) within a column are not significantly different according to Duncan’s multiple range test (*p* < 0.05). Coefficient of variation: Root induction—5.30, number of roots per shoot—15.28, mean root length—14.51. Mean of nine replications.

**Table 4:** Carotenoid and tocopherol contents in the leaf tissues of *Mertensia maritima*.

S/No	Carotenoids	Retention Time (min)	Contents (μg g^-1^ FW)
1	All-*E*-violaxanthin	13.176	5.59 ± 0.31
2	9-*Z*-neoxanthin	14.058	3.44 ± 0.17
3	All-*E*-lutein	18.773	18.49 ± 0.74
4	All-*E*-zeaxanthin	20.364	0.29 ± 0.04
5	All-*E*-β-carotene	30.665	6.42 ± 0.86
6	(*Z*)-β-carotene	31.951	0.74 ± 0.08
7	Total carotenoids	-	34.97 ± 2.12
8	α-tocopherol	14.504	3.82 ± 0.13

Data represent the mean ± standard deviation of three replicates. Carotenoids and α-tocopherol were detected at 450 and 295 nm, respectively. FW: fresh weight.

**Table 5:** Fatty acid composition in the leaf tissues of *Mertensia maritima*.

Retention Time (min)	FAME	% Composition
11.42	C12:0 (Lauric, SFA)	2.44
22.195	C16:0 (Palmitic, SFA)	22.66
25.755	C18:3n6 (γ-Linolenic, PUFA)	14.05
25.89	C18:4n3 (Stearidonic, PUFA)	6.04
26.135	C18:2n6c (Linoleic, PUFA)	16.90
26.29	C18:3n3 (α-Linolenic, PUFA)	30.37
26.89	C18:0 (Stearic, SFA)	3.14
31.2	C20:0 (Arachidic, SFA)	0.84
35.185	C22:0 (Behenic, SFA)	1.12
40.12	C24:0 (Lignoceric, SFA)	2.44
	Total SFAs	32.64
	Total PUFAs	67.36
	PUFAs: SFAs	2.06
	Total lipids (% DW)	10.90

Values are percentages of the total fatty acids, from an average of three extractions and analyses. SFA: Saturated fatty acids; PUFA: Polyunsaturated fatty acids. FAME: fatty acid methyl ester; DW: dry weight.
